# Lung morphology impacts the association between ventilatory variables and mortality in patients with acute respiratory distress syndrome

**DOI:** 10.1186/s13054-023-04350-8

**Published:** 2023-02-13

**Authors:** Hui Chen, Qin Sun, Yali Chao, Yue Liu, Qian Yu, Jianfeng Xie, Chun Pan, Ling Liu, Yi Yang, Haibo Qiu

**Affiliations:** 1 Jiangsu Provincial Key Laboratory of Critical Care Medicine, Department of Critical Care Medicine, Zhongda Hospital, School of Medicine, Southeast University, No. 87, Dingjiaqiao Road, Gulou District, Nanjing, 210009 People’s Republic of China; 2 Department of Critical Care Medicine, The First Affiliated Hospital of Soochow University, Soochow University, No. 899 Pinghai Road, Suzhou, 215000 People’s Republic of China; 3 Department of Intensive Care Unit, Affiliated Hospital of Xuzhou Medical University, Xuzhou Medical University, Xuzhou, 221003 People’s Republic of China; 4 Department of Radiology, Zhongda Hospital, School of Medicine, Southeast University, No. 87, Dingjiaqiao Road, Gulou District, Nanjing, 210009 People’s Republic of China

**Keywords:** Acute respiratory distress syndrome, Ventilatory variables, Lung morphology, 28-day mortality

## Abstract

**Background:**

Acute respiratory distress syndrome (ARDS) patients with different lung morphology have distinct pulmonary mechanical dysfunction and outcomes. Whether lung morphology impacts the association between ventilatory variables and mortality remains unclear. Moreover, the impact of a novel combined ventilator variable [(4×DP) + RR] on morality in ARDS patients needs external validation.

**Methods:**

We obtained data from the Chinese Database in Intensive Care (CDIC), which included adult ARDS patients who received invasive mechanical ventilation for at least 24 h. Patients were further classified into two groups based on lung morphology (focal and non-focal). Ventilatory variables were collected longitudinally within the first four days of ventilation. The primary outcome was 28-day mortality. Extended Cox regression models were employed to explore the interaction between lung morphology and longitudinal ventilatory variables on mortality.

**Findings:**

We included 396 ARDS patients with different lung morphology (64.1% non-focal). The overall 28-day mortality was 34.4%. Patients with non-focal lung morphology have more severe and persistent pulmonary mechanical dysfunction and higher mortality than those with focal lung morphology. Time-varying driving pressure (DP) was more significantly associated with 28-day mortality in patients with non-focal lung morphology compared to focal lung morphology patients (P for interaction = 0.0039). The impact of DP on mortality was more significant than that of respiratory rate (RR) only in patients with non-focal lung morphology. The hazard ratio (HR) of mortality for [(4×DP) + RR] was significant in patients with non-focal lung morphology (HR 1.036, 95% CI 1.027–1.045), not in patients with focal lung morphology (HR 1.019, 95% CI 0.999–1.039).

**Interpretation:**

The association between ventilator variables and mortality varied among patients with different lung morphology. [(4×DP) + RR] was only associated with mortality in patients with non-focal lung morphology. Further validation is needed.

**Supplementary Information:**

The online version contains supplementary material available at 10.1186/s13054-023-04350-8.

## Background

Acute respiratory distress syndrome (ARDS) is a dangerous, potentially fatal respiratory condition in which the lungs sustain a serious, widespread injury that diminishes their ability to provide enough oxygen [1]. The short-term mortality ranged from 30 to 50% worldwide [2]. Lung-protective ventilation strategies have demonstrated survival benefits in randomized clinical trials involving patients with ARDS [3, 4], and recent observational studies demonstrated that mitigated driving pressure (DP) and mechanical power might increase the survival of ARDS [5, 6].

ARDS is a heterogeneous syndrome involving different phenotypes with distinct clinical and outcome characteristics. We previously identified three novel ARDS phenotypes with different severity of pulmonary mechanics and found that the association between pulmonary parameters and mortality varied within phenotypes [7]. Lung morphology is another crucial aspect in distinguishing specific ARDS phenotypes. Patients with non-focal ARDS, as assessed by chest computed tomography (CT), have higher mortality and distinct pulmonary mechanics, including lower lung compliance and a more elevated amount of recruitable lung, than patients with focal lung morphology [8–10]. Whether lung morphology impacts the association between ventilatory variables and mortality has never been explored.

Eduardo L.V and colleagues [11] conducted a study based on 4,549 ARDS patients and found that the impact of DP on mortality was four times as large as that of respiratory rate (RR). Accordingly, they created a novel combined ventilator variable [(4×DP) + RR], which was significantly associated with mortality and was comparable as mechanical power. However, these findings need external validation, and whether the findings vary among patients with different lung morphology is still unclear. Besides, a relatively safe threshold value for [(4×DP) + RR] is undefined.

In this study, we aim to investigate whether lung morphology would influence the association between ventilatory variables and mortality. We also independently validated the impact of [(4×DP) + RR] on mortality in patients with different lung morphology in an external cohort.

## Method

### Study design and participants

We conducted a retrospective observational study in which the data were extracted from the Chinses Database in Intensive Care (CDIC). The latest CDIC contains more than 12,000 patients admitted to the Department of Crit Care Medicine, Zhongda Hospital, Southeast University, China, from January 2014 to July 2022. ARDS patients who received mechanical ventilation for at least 24 h and underwent CT chest examinations within three days after the initiation of mechanical ventilation were eligible for inclusion. ﻿The diagnoses of ARDS were consistent with the Berlin definition [1] (detail in supplementary). ﻿We excluded patients younger than 18 years, and we only included the first intensive care unit (ICU) admission of each patient.

The present study was approved by the Research Ethics Commission of Zhongda Hospital Southeast University (2022ZDSYLL385-Y01). STROBE recommendations were followed.

### Data collection

For every patient, demographic data, primary lung injury of ARDS, vital signs, laboratory results and severity of illness within the first 24 h after inclusion were collected. The disease severity was measured by Acute Physiology and Chronic Health Evaluation (APACHE) II score and Sequential Organ Failure Assessment (SOFA) score. The use of extracorporeal membrane oxygenation (ECMO), prone positioning and neuromuscular blockade agents were also collected. Longitudinal data of ventilatory variables and blood gas were collected on Days 0, 1, 2 and 3. Ventilatory variables included RR, tidal volume (*V*_T_) (scaled to predicted body weight (PBW)), minute ventilation (MV), positive end-expiratory pressure (PEEP), peak pressure (*P*_peak_) and plateau pressure (*P*_plat_) were obtained. All ventilatory variables were extracted under controlled mechanical ventilation. For patients who received ventilation in a volume-controlled assist mode, DP was calculated as *P*_plat_ minus PEEP, if not specified, *P*_peak_ was considered equal to P_plat_ in pressure-regulated modes other than pressure support ventilation. PBW was calculated as equal to [50 + 0.91 × (centimeters of height − 152.4)] in males and [45.5 + 0.91 × (centimeters of height − 152.4)] in females. Combined ventilator variables were calculated as follows: Mechanical power was calculated as [0.098 × V_T_ × RR × (P_peak_ − 0.5 × DP)], and entilatory ratio was calculated as [MV × PaCO_2_ / (PBW × 37.5 × 100)].

The data in CDIC were extracted from the ICU Patient Data Management System which is used to collect patient health information, measurements of organ function parameters, results of laboratory tests and treatment parameters from ICU admission to discharge. Ventilatory variables were extracted per each time frame of 6 h after the initiation of ventilation. For each 24 h, the time-weighted average variables were calculated as the area under the variables versus the time plot. For other clinical variables, we recorded the most abnormal value if a variable was recorded more than once. We defined DP > 35 cmH_2_0, RR > 60 (breaths min-1) and V_T_ > 20 ml/Kg PBW as outliners, ﻿because these physiologically improbable values were assumed to be erroneous. Outliers were transformed into missingness.

### Assessment of lung morphology

The lung CT of all patients was collected within three days after the initiation of mechanical ventilation. All lung CT scans were performed in the supine position. One radiologist and two intensivists characterized patients as having focal or non-focal lung morphology [8]. The focal lung morphology was defined as the presence of consolidations localized only in the lower and back parts of the lungs (Additional file 1: Fig. S1).

### Outcomes

The primary outcome in the present study was 28-day mortality. Secondary outcomes included ICU mortality, in-hospital mortality and ventilation-free days in 28 days.

### Statistical analyses

Values are presented as the mean (standard deviation) or median [interquartile range (IQR)] for continuous variables as appropriate and as the total number (percentage) for categorical variables. Comparisons between groups were made using the *X*^2^ test or Fisher’s exact test for categorical variables and Student’s t test or Mann–Whitney U test for continuous variables as appropriate.

We first compared the 28-day mortality of patients with different lung morphology (focal and non-focal) using Kaplan–Meier curves. The dynamic changes of PaO_2_/FiO_2_ ratio, PaCO_2_, DP, RR, mechanical power and the ventilatory ratio between the two groups were also compared using a liner mixed effects model.

Second, we employed extended multivariate Cox proportional hazards regression [12] to assess the interaction effect between lung morphology and longitudinal ventilator variables on mortality. To adjust for baseline disease severity, we constructed a baseline risk model based on clinical relevance and prior knowledge [11] and included ARDS primary risk factor, PaO_2_/FiO_2_ ratio, arterial pH, PaCO_2_ and respiratory system compliance. All subsequent analyses were pre-adjusted for the baseline risk model. Three Cox regression models (Model A to Model C) containing different ventilator variables were performed (Additional file 1: Table S1). We treated the longitudinal ventilator variables as time-varying exposures in the model. The interaction effect was assessed by the interaction term (lung morphology x ventilator variables) in each Cox regression model.

Third, to validate whether the impact of DP on mortality was four times as large as that of RR, we used Model D (Additional file 1: Table S1) to compare the effect size of DP and RR on 28-day mortality. We also employed restricted cubic splines to visualize the above association. Model E (Additional file 1: Table S1) was constructed to validate the association between the combined ventilator variable [(4×DP) + RR] and mortality. We identified the lowest [(4×DP) + RR] that corresponded to an adjusted hazard ratio estimate of more than 1.00 from Model E. Model F (Additional file 1: Table S1) was used to compare which variable ([(4×DP) + RR] or mechanical power) had a stronger association with mortality. All the above analyses were performed in the whole population and patients with different lung morphology.

Fourth, to further explore the discriminatory performance of each variable on mortality, we calculated the C-index (﻿equivalent to the area under the receiver operating characteristics (AUROC) curve) of each Cox regression model (except for Model D and Model F). To avoid bias ﻿induced by missing data (Additional file 1: Table S2) in all Cox regression modes, ﻿we used multiple imputations by chained equation (MICE) to account for the missing data (detail in supplementary).

Finally, considering that ECMO could influence the impact of ventilatory variables on mortality, we performed a sensitivity analysis after excluding patients receiving ECMO during the study period.

The p-value was calculated to evaluate the differences between groups, and *P* < 0.05 was considered statistically significant. All statistical analyses were performed using R (version 4.0.3).

## Results

### Patients in study

A total of 396 ARDS patients with different lung morphology were enrolled in the final analysis. The flow diagram of study patients is presented in Additional file 1: Fig. S2. The patients had a median age of 64 years (IQR 52–74 years), and 273 (68.9%) were male. Within the first 24 h of ventilation, the DP was 16 cmH_2_0 (IQR:13–20 cmH_2_0), RR was 25 breaths min^−1^ (IQR: 21–30 breaths min^−1^), mechanical power was 20.2 J/min (IQR: 14.8–25.9 J/min), and the ventilatory ratio was 1.86 (IQR: 1.37–2.43). Pneumonia was the leading cause of ARDS (62.1%). The 28-day all-cause mortality was 34.4%. Additional details are summarized in Table 1.

**Table 1:** Clinical variables and outcomes in ARDS patients with different lung morphology

	All (*n* = 396)	Lung morphology		*P* value
		Focal (*n* = 142)	Non-focal (*n* = 254)	
Age (years)	64 (52, 74)	63.5 (52, 73)	64 (52, 74)	0.92
Male (gender), *n* (%)	273 (68.9)	101 (71.1)	172 (67.7)	0.55
BMI (kg/m^2^)	24.0 (22.0, 26.1)	24.2 (22.9, 27.0)	23.6 (21.1, 25.7)	0.002
ARDS Primary risk factor, *n* (%)				< 0.001
Pneumonia	246 (62.1)	53 (37.3)	193 (76)	
Sepsis	68 (17.2)	45 (31.7)	23 (9.1)	
Aspiration	34 (8.6)	14 (9.9)	20 (7.9)	
Other	48 (12.1)	30 (21.1)	18 (7.1)	
SOFA score	8 (6, 11)	8 (6, 11)	8.5 (6, 11)	0.93
APACHE II score	22.3 (7.6)	21.7 (7.0)	22.5 (7.9)	0.29
Severity of ARDS at baseline, *n* (%)				< 0.001
Mild	123 (31.1)	56 (39.4)	67 (26.4)	
Moderate	203 (51.3)	73 (51.4)	130 (51.2)	
Severe	70 (17.7)	13 (9.2)	57 (22.4)	
Vital signs in the first 24 h				
Heart rate (beats min^−1^)	98 (94, 99)	98 (94.3, 99)	97 (94, 99)	0.12
MAP (mmHg)	69 (65.3, 74.7)	69 (65.7, 76)	68.8 (64.7, 74)	0.14
Temperature (℃)	37.8 (0.96)	37.9 (0.98)	37.7 (0.95)	0.21
Parameters of mechanical ventilation in the first 24 h				
Respiratory rate (breaths min^−1^)	25 (21, 30)	24.5 (20, 28)	25.5 (22, 31)	0.0080
Tidal volume (ml/kg PBW)	8.1 (6.9, 9.6)	8.4 (7.3, 9.8)	7.9 (6.7, 9.3)	0.007
Minute ventilation (L/min)	12.4 (9.5, 15.6)	12.6 (10.0, 15.9)	12.3 (9.4, 15.2)	0.43
PEEP (cmH_2_0)	9 (7, 10)	8 (6, 10)	10 (8, 11)	< 0.001
Peak pressure (cmH_2_0)	24 (21, 28)	23 (20, 26)	25 (21.25, 29)	< 0.001
Driving pressure (cmH_2_0)	16 (13, 20)	15 (13, 18)	16 (13, 20)	0.17
Mechanical power (J/min)	20.2 (14.8, 25.9)	19.9 (14.4, 24.4)	20.3 (14.9, 27.1)	0.11
Compliance (ml/cmH_2_0)	30.9 (22.0, 41.5)	32.2 (25.6, 43.7)	30.3 (20.3, 40.7)	0.017
Ventilatory ratio	1.86 (1.37, 2.43)	1.78 (1.34, 2.24)	1.92 (1.39, 2.51)	0.18
PaCO_2_ (mmHg)	34.8 (29.8, 40.4)	33.9 (28.4, 39.4)	35.3 (30.3, 41.4)	0.018
PaO_2_/FiO_2_ ratio (mmHg)	158.4 (116.5, 213.4)	179.1 (144.9, 237.2)	147.0 (107.6, 203.3)	< 0.001
Laboratory data in the first 24 h				
pH	7.39 (7.32, 7.44)	7.38 (7.32, 7.43)	7.39 (7.32, 7.44)	0.62
BUN	9.6 (6.3, 14.5)	10.7 (6.75, 15.6)	9.35 (5.65, 13.8)	0.048
Creatinine (mmol/L)	97 (68, 155.5)	111.5 (72.25, 181)	91 (65, 143)	0.007
Total bilirubin (μmol/L)	15 (9.3, 26.2)	17.4 (11.6, 28.25)	13.65 (8.47, 23.15)	0.022
D-dimer (ug/ml)	2.25 (0.78, 4.68)	2.58 (0.82, 4.70)	2.10 (0.78, 4.63)	0.44
Bicarbonate (mmol/L)	21.1 (18.3, 24.1)	20.5 (17.8, 23.9)	21.6 (18.5, 24.7)	0.048
Lactate (mmol/L)	1.9 (1.28, 3.1)	2 (1.3, 3.58)	1.8 (1.2, 2.68)	0.021
Use of adjunct treatments				
ECMO, *n* (%)	44 (11.1)	5 (3.5)	39 (15.4)	< 0.001
Prone positioning, *n* (%)	177 (44.7)	25 (17.6)	152 (59.8)	< 0.001
NMBA, *n* (%)	95 (24.0)	5 (3.5)	90 (35.4)	< 0.001
Clinical outcomes				
Alive and VFDs at Day 28 (days)	7.0 (0, 21.0)	15.5 (0, 22.4)	0 (0, 19.4)	< 0.001
ICU mortality, *n* (%)	111 (28)	21 (14.8)	90 (35.4)	< 0.001
Hospital mortality, *n* (%)	119 (30.1)	26 (18.3)	93 (36.6)	< 0.001
28-day mortality, *n* (%)	136 (34.3)	28 (19.7)	108 (42.5)	< 0.001

*BMI* body mass index, *ARDS* acute respiratory distress syndrome, *SOFA* sequential organ failure assessment, *APACHE* acute physiology and chronic health evaluation II, *MAP* mean arterial blood, *PBW* predicted body weight, *PEEP* positive end-expiratory pressure, *PaCO*_2_ partial pressure of Carbon Dioxide, *PaO*_2_ partial pressure of oxygen (pressure), *BUN* blood urea nitrogen, *ECMO* extracorporeal membrane oxygenation, *NMBA* neuromuscular blockade agents, *VFD* ventilator-free days, *ICU* intensive care unit

### Comparison between patients with focal and non-focal lung morphology

**Fig. 1:**
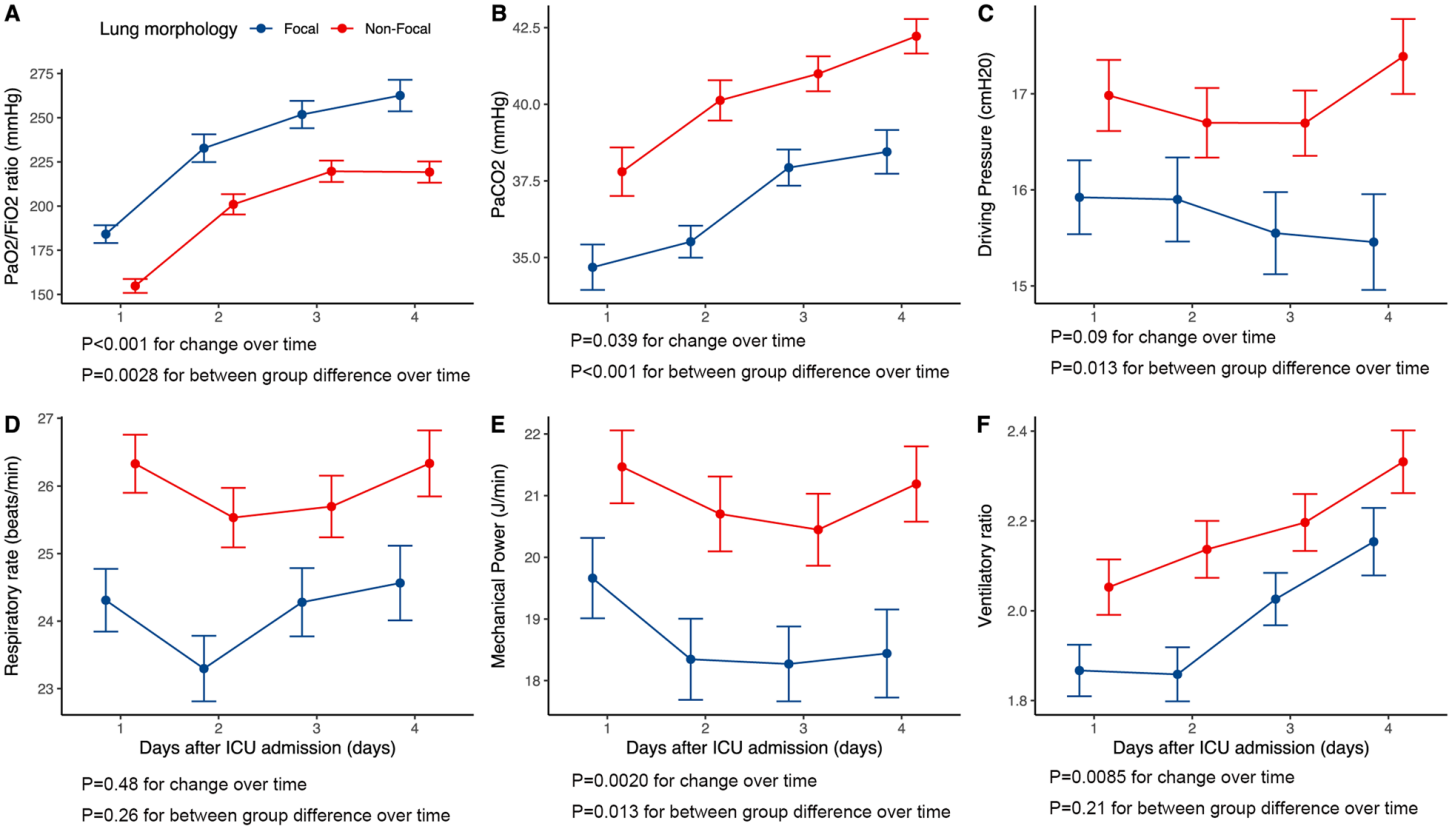
Temporal changes of ventilatory variables between patients with focal and non-focal lung morphology within the first four days of mechanical ventilation. Figure shows temporal changes in PaO_2_/FiO_2_ ratio (**A**), PaCO_2_ (**B**), Driving pressure (**C**), Respiratory rate (**D**), Mechanical Power (**E**) and Ventilatory ratio (**F**)

Of the study cohort, 254 patients (64.1%) were classified as having non-focal lung morphology, and the remaining were focal lung morphology (*n* = 142). The comparison between focal and non-focal lung morphology is presented in Table 1 and Additional file 1: Fig. S3-S5. Patients in the non-focal lung morphology group had a significantly lower PaO_2_/FiO_2_ ratio, respiratory compliance_,_ and a higher RR within the first 24 h of ventilation than those in the focal lung morphology group. In the liner mixed effects model, most ventilatory variables are changed dynamically during the first four days of mechanical ventilation, and there was a significant difference between the two groups regarding dynamic changes of PaO_2_/FiO_2_ ratio, PaCO_2_, DP and mechanical power (Fig. 1).

The 28-day mortality rate was significantly higher in patients with non-focal lung morphology than those with focal lung morphology (42.5% vs. 19.7%, *P* < 0.001). Kaplan–Meier survival curves also showed that the 28-day mortality was higher in the non-focal lung morphology group (Additional file 1: Fig. S6).

### Interaction effect between lung morphology and ventilator variables on mortality

**Fig. 2:**
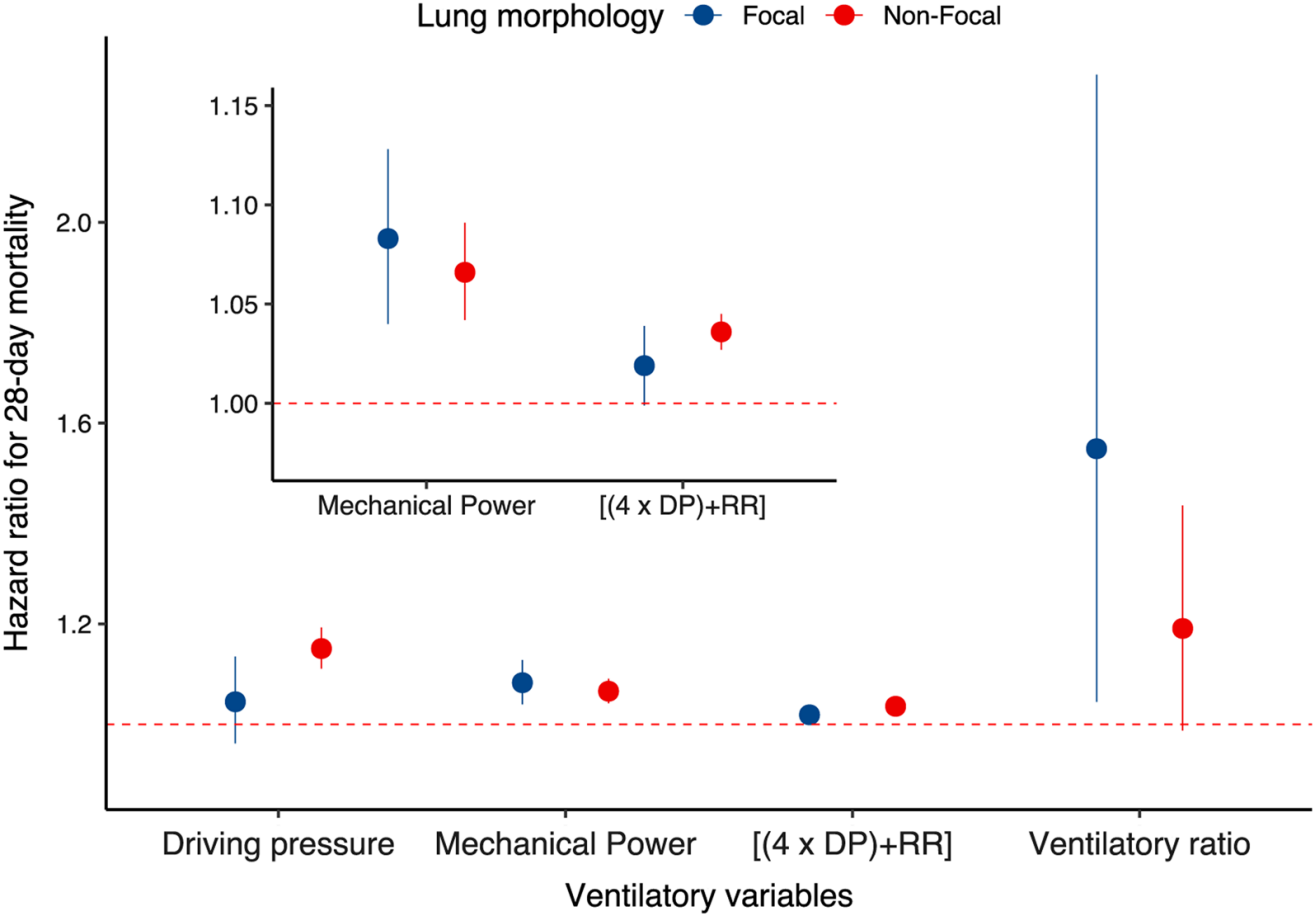
The association between time-varying ventilatory variables and 28-day mortality in patients with focal and non-focal lung morphology. DP: Driving pressure; RR: Respiratory rate

After adjusting for the baseline risk model, time-varying DP was more significantly associated with 28-day mortality in patients with non-focal lung morphology compared to patients with focal lung morphology (P for interaction = 0.0039). Time-varying mechanical power was associated with increased 28-day mortality in patients with focal and non-focal lung morphology. Although the association between time-varying ventilatory ratio and 28-day mortality seemed to be stronger among patients with focal lung morphology, no significant interaction was detected (P for interaction = 0.25) (Table 2 and Fig. 2).

**Table 2:** Association between time-varying ventilatory variables and mortality in ARDS patients with different lung morphology

Model	Lung morphology				*P* for interaction
	Focal (*n* = 142)		Non-focal (*n* = 254)		
	HR (95% CI)	*P* value	HR (95% CI)	*P* value	
Model A					
Driving pressure	1.045 (0.962–1.135)	0.30	1.151 (1.111–1.193)	< 0.001	0.0039
Model B					
Mechanical Power	1.083 (1.04–1.128)	< 0.001	1.066 (1.042–1.091)	< 0.001	0.31
Model C					
Ventilatory ratio	1.549 (1.045–2.294)	0.029	1.191 (0.988–1.436)	0.066	0.25
Model D*					
Driving pressure	1.060 (0.985–1.142)	0.12	1.156 (1.116–1.199)	< 0.001	–
Respiratory rate	1.096 (1.030–1.167)	0.0041	1.027 (1.002–1.053)	0.033	–
Model E					
[(4 × DP) + RR]	1.019 (0.999–1.039)	0.057	1.036 (1.027–1.045)	< 0.001	0.093
Model F ([(4 × DP) + RR] vs. Power)^#^					
Mechanical Power	1.079 (1.029–1.131)	0.0017	1.034 (1.012–1.057)	0.0026	–
[(4 × DP) + RR]	1.004 (0.983–1.025)	0.74	1.030 (1.020–1.040)	< 0.001	–

*ARDS* acute respiratory distress syndrome, *DP* driving pressure, *RR* respiratory rate, *HR* hazard ratio, *CI* confidence interval

*Model D was employed to compare the effect size of DP and RR on 28-day mortality in two groups, and interaction P value was not calculated

^#^The interaction effect between lung morphology and power or [(4 × DP) + RR] on 28-day mortality was assessed in Model B and Model E, respectively. Model F was aimed to assess which variable had a stronger association with mortality in two groups, and interaction P value was not calculated

### Validation of the association between [(4×DP) + RR] and 28-day mortality

**Fig. 3:**
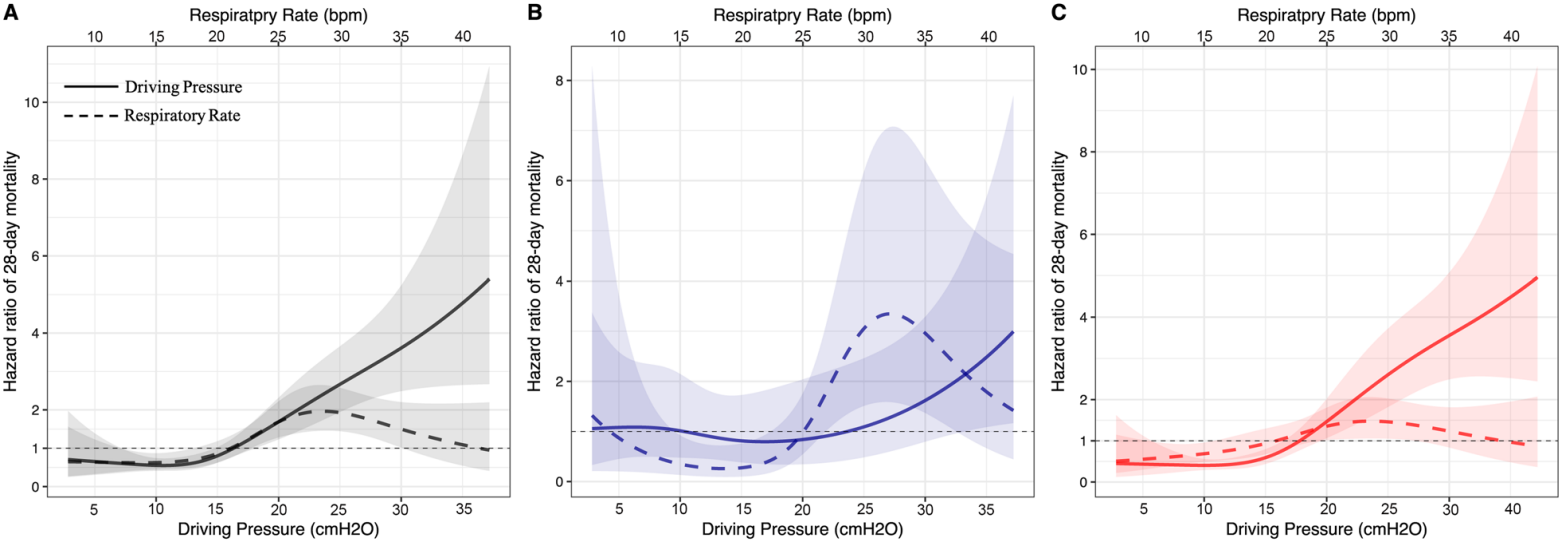
The hazard ratio of 28-day mortality for driving pressure and respiratory rate in whole patients (**A**), patients with focal lung morphology (**B**) and patients with non-focal lung morphology (**C**)

In Model D, we found that both DP and RR were associated with 28-day mortality, and the effect size of each 1 cmH_2_0 increase in DP was larger than that of each 1 breath/min increase in RR in the whole population (approximately 2.8 times), while these associations only remained in patients with non-focal lung morphology (approximately 4.6 times) (Fig. 3 and Additional file 1: Table S3).

**Fig. 4:**
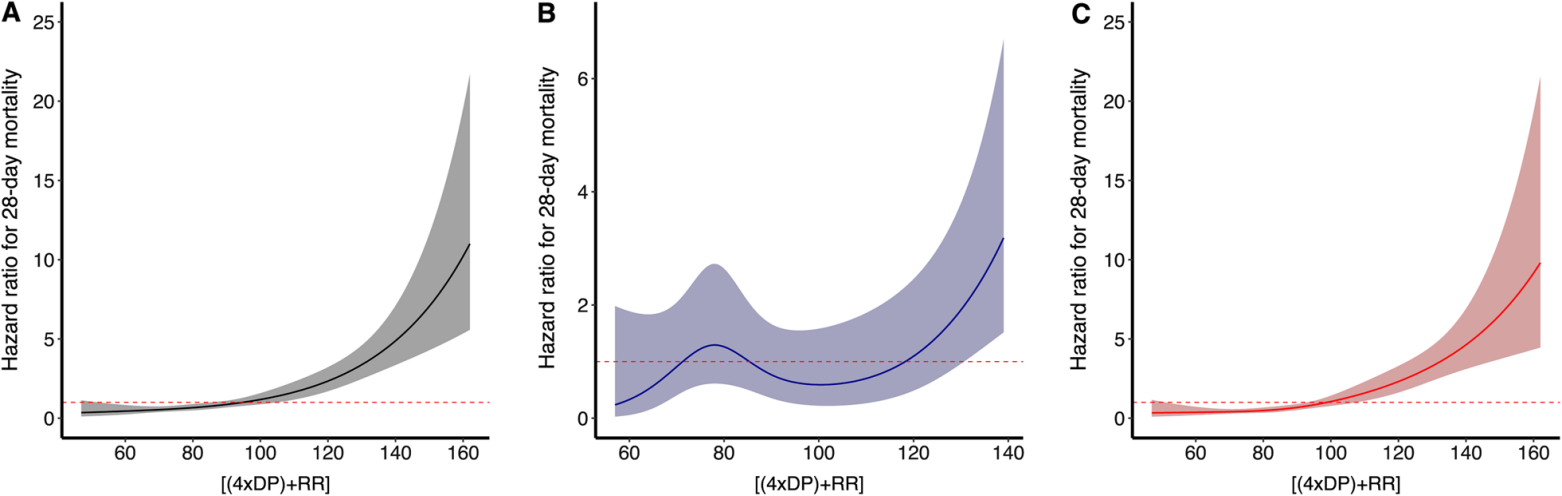
The association between [(4×DP) + RR] and 28-day mortality in whole patients (**A**), patients with focal lung morphology (**B**) and patients with non-focal lung morphology (**C**)

In Model E, the combined ventilator variable [(4×DP) + RR] was significantly associated with mortality in whole patients and patients with non-focal lung morphology, but not in patients with focal lung morphology, the interaction p value was 0.093 (Fig. 4 and Table 2). After including mechanical power and [(4×DP) + RR] in the same model (Model F), [(4×DP) + RR] remained a significant predictor of mortality in patients with non-focal lung morphology (Table 2). This analysis was consistent with a comparable discriminatory performance of [(4×DP) + RR] as compared to mechanical power in the non-focal group (Additional file 1: Fig. S7).

The lowest [(4×DP) + RR], during the first four days of mechanical ventilation, that was associated with an adjusted HR of more than 1.00 for 28-day mortality was 95 in the whole patients and 99 in patients with non-focal lung morphology (Fig. 4).

### Sensitivity analysis

The results of the sensitivity analysis were generally consistent with the primary analysis (Additional file 1: Table S4). Specifically, both DP and [(4×DP) + RR] were more significantly associated with 28-day mortality in patients with non-focal lung morphology compared to patients with focal lung morphology.

## Discussion

The novel findings of our study can be summarized as follows. First, patients with non-focal lung morphology have more severe and persistent pulmonary mechanical dysfunction and higher mortality than those with focal lung morphology. Second, lung morphology significantly impacts the association between time-varying driving pressure and mortality. Third, only in patients with non-focal lung morphology, the impact of DP on mortality was more significant than that of RR, and the combined ventilator variable [(4×DP) + RR] was comparable to mechanical power in predicting mortality.

Consistent with previous research, patients with non-focal lung morphology were distinct from patients with focal lung morphology in clinical and outcomes characteristics. Luciano Gattinoni and colleagues [9] grouped patients into lower (similar to focal lung morphology) and higher (similar to non-focal lung morphology) percentages of potentially recruitable lung based on quantitative analysis of lung CT at 5 and 45 cmH_2_0 PEEP levels, and concluded that patients with a higher percentage of potentially recruitable lung had a lower PaO_2_/FiO_2_ ratio, respiratory compliance and a higher PaCO_2_, dead space and mortality rate than those in the group with a lower percentage of the potentially recruitable lung. Additionally, our study also detected that the differences in most respiratory variables between groups persisted through the first four days of mechanical ventilation.

The association between ventilatory variables and mortality among patients with different lung morphology was not always in accordance. There are several explanations for our results. First, patients in the focal lung morphology group were accomplished with less pulmonary mechanical dysfunction (higher DP) but more extra-pulmonary dysfunction (more elevated creatinine, total bilirubin and lactate), suggesting that patients with focal lung morphology have worse outcomes independently from DP, but may dependently from other makers of organ dysfunction [13]. Second, mechanical power is composed of multiple respiratory variables, which are both mathematically and physiologically coupled [14]. In focal lung morphology patients, RR was significantly associated with mortality, whereas DP was the opposite, which could explain, in part, the significant effect of mechanical power on mortality in patients with different lung morphology. Finally, since our study was a retrospective cohort study, important predictors could have insignificant associations ascribed to other measured or unmeasured confounders. More external validations are needed.

Gattinoni and colleagues first demonstrated mechanical power as a compound indicator in ventilator-induced lung injury (VILI). They found that the same percentage increase of DP and RR can produce an identical exponential increase of mechanical power, with an exponent of 2.0 and 1.4, respectively [15]. Recently, a muti-database study identified that the impact of DP on mortality was four times as large as that of RR in ARDS patients and derived a novel ventilator variable [(4×DP) + RR] accordingly, which was as informative as mechanical power and easier to assess at the bedside [11]. These findings were validated in our study, but only in patients with non-focal lung morphology, and the times was 4.6. Whether the combined variable [(4×DP) + RR] is applicable depends on the different weights of DP and RR on mortality. In patients with focal lung morphology, RR was valuable while DP was not, the effects of RR on mortality could be counteracted by [4×DP] and therefore [(4×DP) + RR] was insignificant. Additionally, whether “four times” was generalized in all ARDS remains uncertain and needs more prospective and extensive sample size studies.

There was a different impact between mechanical power with [(4×DP) + RR] on mortality in the focal lung morphology group. Mechanical power was consisting of elastic dynamic, elastic static and resistive power, which represents the energy transferred from the ventilator to the respiratory system essentially [15], regardless of the lung morphology. Whereas the [(4×DP) + RR] could only reflect elastic dynamic (related to DP), and the value of DP in the focal lung morphology group is limited as we discussed above.

The current study has some implications for clinical practice. For patients with non-focal lung morphology, ventilatory strategies including high respiratory rate and relatively low *V*_T_ may be beneficial. The upper limit of RR can be calculated based on the threshold value of [(4×DP) + RR]. In contrast, patients with focal lung morphology may benefit more from a relatively high *V*_T_, low respiratory rate and early prone position [16, 17]. The survival benefit of these individual ventilator settings has been proved in ARDS patients after accounting for the misclassification of lung morphology [18].

Several limitations in the present study should be considered. First, the present study was a retrospective observational study based on a single center, the sample size was limited, and we considered only segmental measured confounders, the residual measured confounders and unmeasured confounders cannot be fully included, such as the prone position ventilation, use of neuromuscular blockade agents. Second, although the lung morphologies were classified as focal and non-focal by one radiologist and two intensivists, we cannot exclude the possibility of misclassification of lung morphology. Additionally, the whole lung CT was only collected once within three days after initiation of mechanical ventilation, we can only assume that the lung morphology would not change significantly during the early phase of ventilation. Third, DP was calculated as P_peak_ minus PEEP in some patients, which may lead to underestimating mechanical power. In addition, the intrinsic PEEP was limited in the present study, in the presence of intrinsic PEEP, the ideal combination of RR and DP might be ﻿unattainable [19]. Finally, we identified the threshold of [(4×DP) + RR] based on an increased risk of death at 28 days. Give the single-center and limited sample size; the threshold may not always be applicable in other patients. Long-term outcomes of ARDS also be influenced by the high intensity of mechanical ventilation [20], while the threshold of [(4×DP) + RR] for long-term outcomes may be different from that we have identified.

## Conclusion

In conclusion, lung morphology impacts the association between ventilatory variables and mortality in ARDS patients, especially for driving pressure. The combined ventilator variable [(4×DP) + RR] was informative in patients with non-focal lung morphology, not in patients with focal lung morphology.

## Supplementary Information

**Additional file 1**: **Table S1**. Different multivariate Cox proportional hazards regression models; **Table S2**. Percentage of missing data in the variables of interest at baseline; **Table S3**. The impact of Driving pressure and respiratory rate on 28-day mortality in Model D; **Table S4**. Association between time-varying ventilatory variables and mortality in ARDS patients with different lung morphology after excluding patients receiving ECMO (n=352); **Fig. S1**. Lung morphology of ARDS patients based on lung CT: non-focal lung morphology (**A**); focal lung morphology (**B**); **Fig. S2** patients selection in the CDIC cohort. **Fig. S3**. The distribution of ventilatory variables between patients with focal lung morphology and patients with non-focal lung morphology; **Fig. S4**. The correlation between Driving pressure and PF ratio (**A**), respiratory compliance (**B**), Tidal volume (**C**) and [(4×DP)+RR] (**D**) in patients with different lung morphology; **Fig. S5**. The correlation between mechanical power and PF ratio (**A**), Respiratory compliance (**B**), tidal volume (**C**) and [(4×DP)+RR] (**D**) in patients with different lung morphology; **Fig. S6**. Mortality by lung morphology in patients with ARDS; **Fig. S7**. The C-index of each Cox regression Model in total patients and patients with different with lung morphology (DOCX 8.7M).

## Data Availability

For data in CDIC cohort, data are available upon reasonable request and with the approval from the Department of Critical Care Medicine, Zhongda Hospital, School of Medicine, Southeast University.

## Abbreviations

ARDS: Acute respiratory distress syndrome
DP: Driving pressure
CT: Computed tomography
RR: Respiratory rate
ICU: Intensive care unit
APACHE II: Acute Physiology and Chronic Health Evaluation
SOFA: Sequential Organ Failure Assessment
V_T_: Tidal volume
PBW: Predicted body weight
MV: Minute ventilation
PEEP: Positive end-expiratory pressure
P_peak_: Peak pressure
P_plat_: Plateau pressure
AUROC: Area under the receiver operating characteristics curve
MICE: Multiple imputations by chained equation
ECMO: Extracorporeal membrane oxygenation
VILI: Ventilator-induced lung injury
