# Supplementary material for: Lung morphology impacts the association between ventilatory variables and mortality in patients with acute respiratory distress syndrome
Source: Crit Care. 2023 Feb 13;27:59. doi: 10.1186/s13054-023-04350-8 (PMC9926837; doi:10.1186/s13054-023-04350-8)
Supplement: Supplementary file 1 — Additional file 1: Table S1. Different multivariate Cox proportional hazards regression models; Table S2. Percentage of missing data in the variables of interest at baseline; Table S3. The impact of Driving pressure and respiratory rate on 28-day mortality in Model D; Table S4. Association between time-varying ventilatory variables and mortality in ARDS patients with different lung morphology after excluding patients receiving ECMO (n=352); Fig. S1. Lung morphology of ARDS patients based on lung CT: non-focal lung morphology (A); focal lung morphology (B); Fig. S2 patients selection in the CDIC cohort. Fig. S3. The distribution of ventilatory variables between patients with focal lung morphology and patients with non-focal lung morphology; Fig. S4. The correlation between Driving pressure and PF ratio (A), respiratory compliance (B), Tidal volume (C) and [(4×DP)+RR] (D) in patients with different lung morphology; Fig. S5. The correlation between mechanical power and PF ratio (A), Respiratory compliance (B), tidal volume (C) and [(4×DP)+RR] (D) in patients with different lung morphology; Fig. S6. Mortality by lung morphology in patients with ARDS; Fig. S7. The C-index of each Cox regression Model in total patients and patients with different with lung morphology (DOCX 8.7M). [file 13054_2023_4350_MOESM1_ESM.docx]

**Additional methods:**

**A: Approach to identify ARDS patients**

To identify ARDS patients in CDIC cohort, we first extracted all acute respiratory failure patients with mechanical ventilation from the database, then medical history, radiographs (lung CT or chest X-ray), blood gases and PEEP were reviewed by experienced intensivists and radiologist, and patients were diagnosed with ARDS if they met the Berlin definition. If there is a dispute, the diagnosis was determined by a more experienced intensivist.

B: **Approach to missing Data**

Missing data were summarized in Table S2. Prior to each Cox regression model, we assumed that missing data was “missing at random”, We used multiple imputation by chained equation (MICE) which generated values for all missing data using the observed data for all patients. In total we imputed 5 different data sets (n = 5), the imputation method was weighted predictive mean matching. We used ‘mice’ package in R studio to impute the data (https://cran.rproject.org/web/packages/mice).

**Table S1: Different multivariate Cox proportional hazards regression models**

| Model A | DP was added to the Baseline Risk Model. |
| --- | --- |
| Model B | Mechanical power was added to the Baseline Risk Model. |
| Model C | Ventilatory ratio was added to the Baseline Risk Model. |
| Model D | DP and RR were simultaneously added to the Baseline Risk Model, to compare the effect size of DP and RR on 28-day mortality |
| Model E | Based on previous research, a combination of RR and DP computed as [(4xDP) +RR] was added to the Baseline Risk Model. |
| Model F | The combined variable [(4xDP) +RR] and total power were simultaneously added to the Baseline Risk Model, to assess which variable had a stronger association with mortality |

**Table S2:** **Percentage of missing data in the variables of interest at baseline**

|  | n (%) |
| --- | --- |
| Age (years) | 0 (0%) |
| Male (gender) | 0 (%) |
| BMI (kg/m^2^) | 5 (1.3%) |
| ARDS Primary risk factor | 0 (%) |
| SOFA score | 105 (26.5%) |
| APACHE II score | 50 (14.1%) |
| Severity of ARDS at baseline | 0 (%) |
| Heart rate (beats min^-1^) | 4 (1.0%) |
| MAP (mmHg) | 9 (2.3%) |
| Temperature (℃) | 19 (4.8%) |
| Respiratory rate (breaths min^-1^) | 12 (3.0%) |
| Tidal volume (ml/kg PBW) | 3 (0.8%) |
| Minute ventilation (L/min) | 12 (3.0%) |
| PEEP (cmH_2_0) | 22 (5.6%) |
| Peak pressure (cmH_2_0) | 20 (5.1%) |
| Driving pressure (cmH_2_0) | 24 (6.1%) |
| Mechanical power (J/min) | 32 (8.1%) |
| Compliance (ml/cmH_2_0) | 24 (6.1%) |
| Ventilatory ratio | 16 (4.0%) |
| PaCO_2_ (mmHg) | 0 (%) |
| PaO_2_/FiO_2_ ratio (mmHg) | 0 (%) |
| pH | 0 (%) |
| BUN | 43 (10.9%) |
| Creatinine (mmol/L) | 29 (7.3%) |
| Total bilirubin (μmol/L) | 221 (55.8%) |
| D-dimer (ug/ml) | 35 (8.8%) |
| Bicarbonate (mmol/L) | 0 (%) |
| Lactate (mmol/L) | 0 (%) |

BMI: body mass index, ARDS: acute respiratory distress syndrome, SOFA: sequential organ failure assessment, APACHE: acute physiology and chronic health evaluation II, MAP: mean arterial blood, PBW: predicted body weight, PEEP: positive end-expiratory pressure, PaCO_2_: partial pressure of Carbon Dioxide, PaO_2_: partial pressure of oxygen, pressure, BUN: blood urea nitrogen.

Table S3: The impact of Driving pressure and respiratory rate on 28-day mortality in Model D.

|  | Total (n=396) | | Focal lung morphology (n=142) | | Non-Focal lung morphology (n=254) | |
| --- | --- | --- | --- | --- | --- | --- |
|  | Coefficient (se) | P value | Coefficient (se) | P value | Coefficient (se) | P value |
| Driving Pressure | 0.124 (0.016) | <0.001 | 0.060 (0.038) | 0.11 | 0.143 (0.018) | <0.001 |
| Respiratory rate | 0.045 (0.011) | <0.001 | 0.092 (0.032) | 0.0041 | 0.031 (0.013) | 0.014 |
| Arterial pH, each 0.1 | -0.033 (0.120) | 0.78 | 0.013 (0.290) | 0.97 | 0.013 (0.139) | 0.93 |
| PaO_2_/FiO_2_ ratio, each 10 mmHg | -0.861 (0.244) | 0.00042 | -0.827 (0.616) | 0.18 | -0.780 (0.283) | 0.0048 |
| PaCO_2_, each 5 mmHg | -0.742 (0.327) | 0.023 | -0.777 (0.727) | 0.28 | -0.764 (0.399) | 0.055 |
| Respiratory compliance, each 5 ml/cmH_2_0 | -0.328 (0.327) | 0.31 | -0.395 (0.682) | 0.56 | -0.334 (0.372) | 0.37 |
| ARDS Primary risk factor | | | | | | |
| Pneumonia | Reference | -- | Reference | -- | Reference | -- |
| Sepsis | 0.171 (0.266) | 0.64 | 0.710 (0.431) | 0.099 | 0.239 (0.336) | 0.48 |
| Aspiration | 0.059 (0.357) | 0.87 | -0.782 (0.957) | 0.41 | 0.484 (0.384) | 0.21 |
| Other | -0.576 (0.306) | 0.060 | -0.505 (0.622) | 0.42 | -0.367 (0.373) | 0.32 |

PaO_2_: partial pressure of oxygen, pressure; PaCO_2_: partial pressure of Carbon Dioxide; ARDS: acute respiratory distress syndrome.

**Table S4: Association between time-varying ventilatory variables and mortality in ARDS patients with different lung morphology after excluding patients receiving ECMO (n=352).**

| **Model** | **Lung morphology** | | | | **P for interaction** |
| --- | --- | --- | --- | --- | --- |
|  | **Focal (n=137)** | | **Non-Focal (n=215)** | |  |
|  | HR (95% CI) | P value | HR (95% CI) | P value |  |
| **Model A** | | | | | |
| Driving pressure | 1.057 (0.974-1.148) | 0.19 | 1.155 (1.109-1.203) | <0.001 | 0.042 |
| **Model B** | | | | | |
| Mechanical Power | 1.093 (1.052-1.135) | <0.001 | 1.076 (1.048-1.105) | <0.001 | 0.40 |
| **Model C** | | | | | |
| Ventilatory ratio | 1.614 (1.088-2.393) | 0.017 | 1.205 (0.972-1.494) | 0.089 | 0.17 |
| **Model D*** | | | | | |
| Driving pressure | 1.073 (0.997-1.154) | 0.058 | 1.158 (1.111-1.206) | <0.001 | — |
| Respiratory rate | 1.112 (1.048-1.180) | <0.001 | 1.034 (1.004-1.064) | 0.024 | — |
| **Model E** | | | | | |
| [(4 x DP)+RR] | 1.022 (0.998-1.039) | 0.059 | 1.037 (1.027-1.047) | <0.001 | 0.11 |
| **Model F ([(4 x DP)+RR] vs Power)^#^** | | | | | |
| Mechanical Power | 1.085 (1.035-1.137) | <0.001 | 1.043 (1.016-1.071) | 0.0015 | — |
| [(4 x DP)+RR] | 1.006 (0.985-1.027) | 0.59 | 1.030 (1.018-1.041) | <0.001 | — |

ARDS: acute respiratory distress syndrome; ECMO: extracorporeal membrane oxygenation; DP: driving pressure; RR: respiratory rate; HR: hazard ratio; CI: confidence interval.

^*^Model D was employed to compare the effect size of DP and RR on 28-day mortality in two groups, and interaction P value was not calculated.

^#^The interaction effect between lung morphology and power or [(4 x DP)+RR] on 28-day mortality was assessed in Model B, and Model E, respectively. Model F was aimed to assess which variable had a stronger association with mortality in two groups, and interaction P value was not calculated.

Figure S1: Lung morphology of ARDS patients based on lung CT: Non-focal lung morphology (A); Focal lung morphology (B).


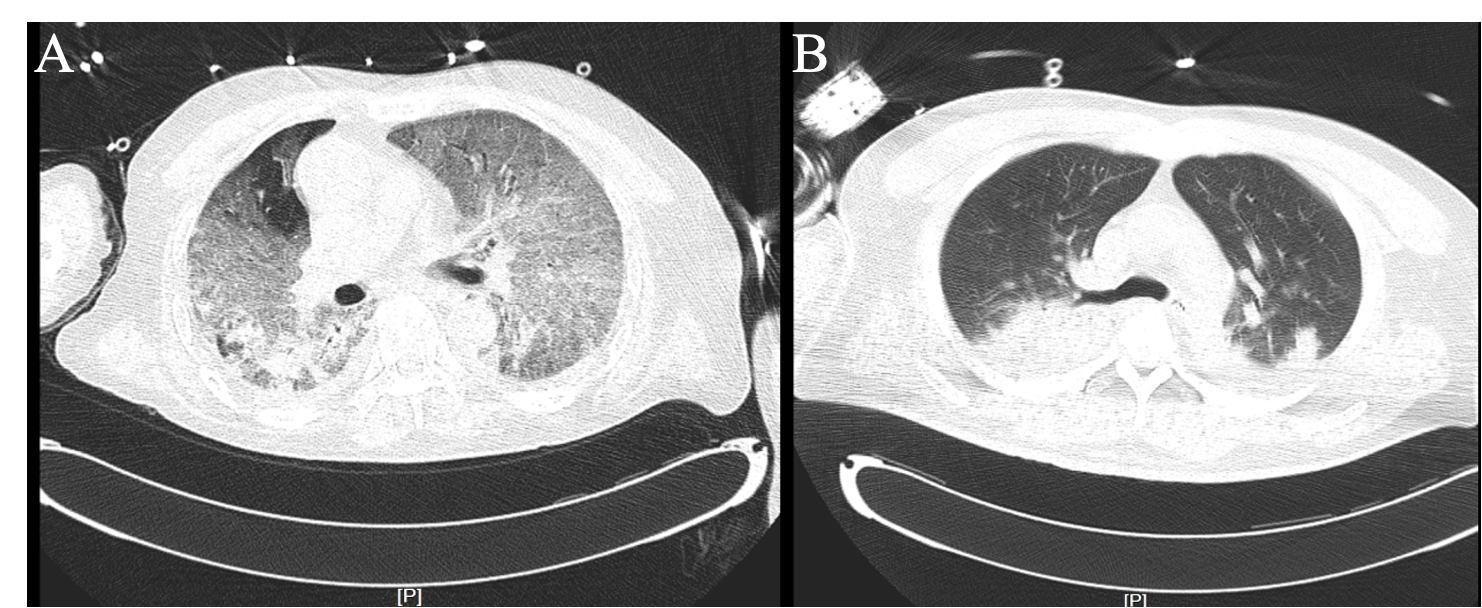


Figure S2: Patients selection in the CDIC cohort.

Figure S3: The distribution of ventilatory variables between patients with focal lung morphology and patients with non-focal lung morphology.


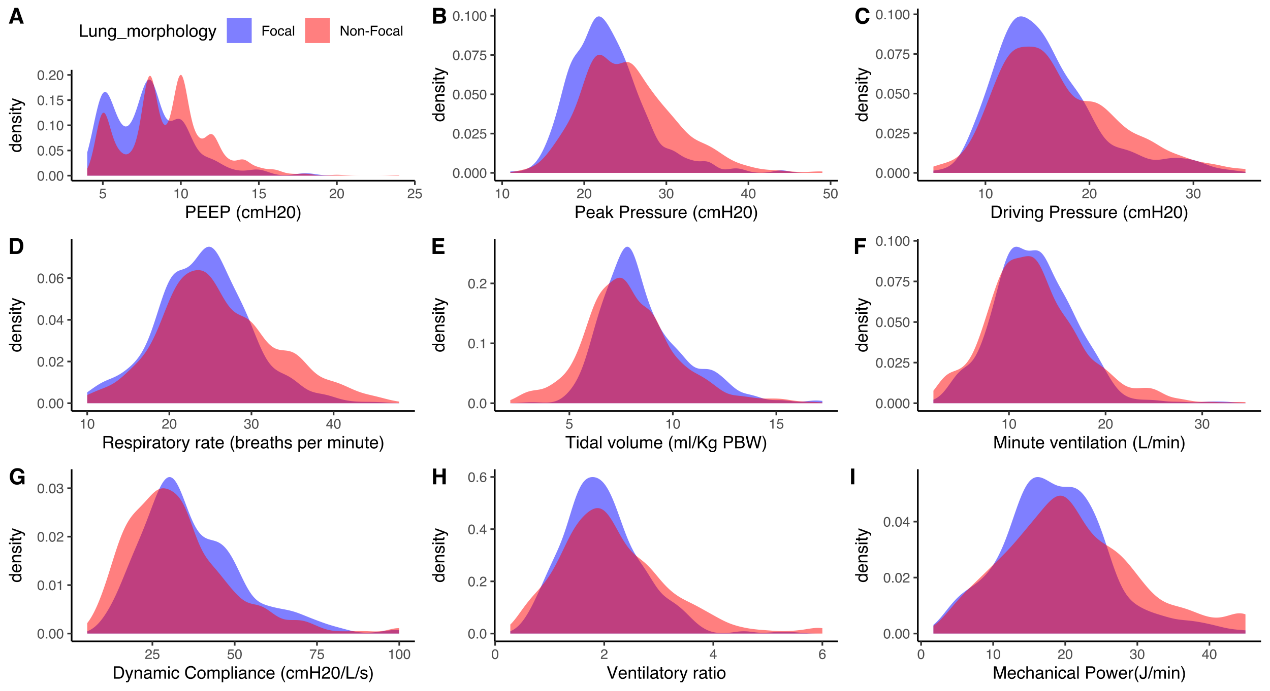


PEEP: positive end-expiratory pressure.

Figure S4: The Correlation between Driving pressure and PF ratio (A), Respiratory compliance (B), Tidal volume (C) and [(4xDP)+RR] (D) in patients with different lung morphology.


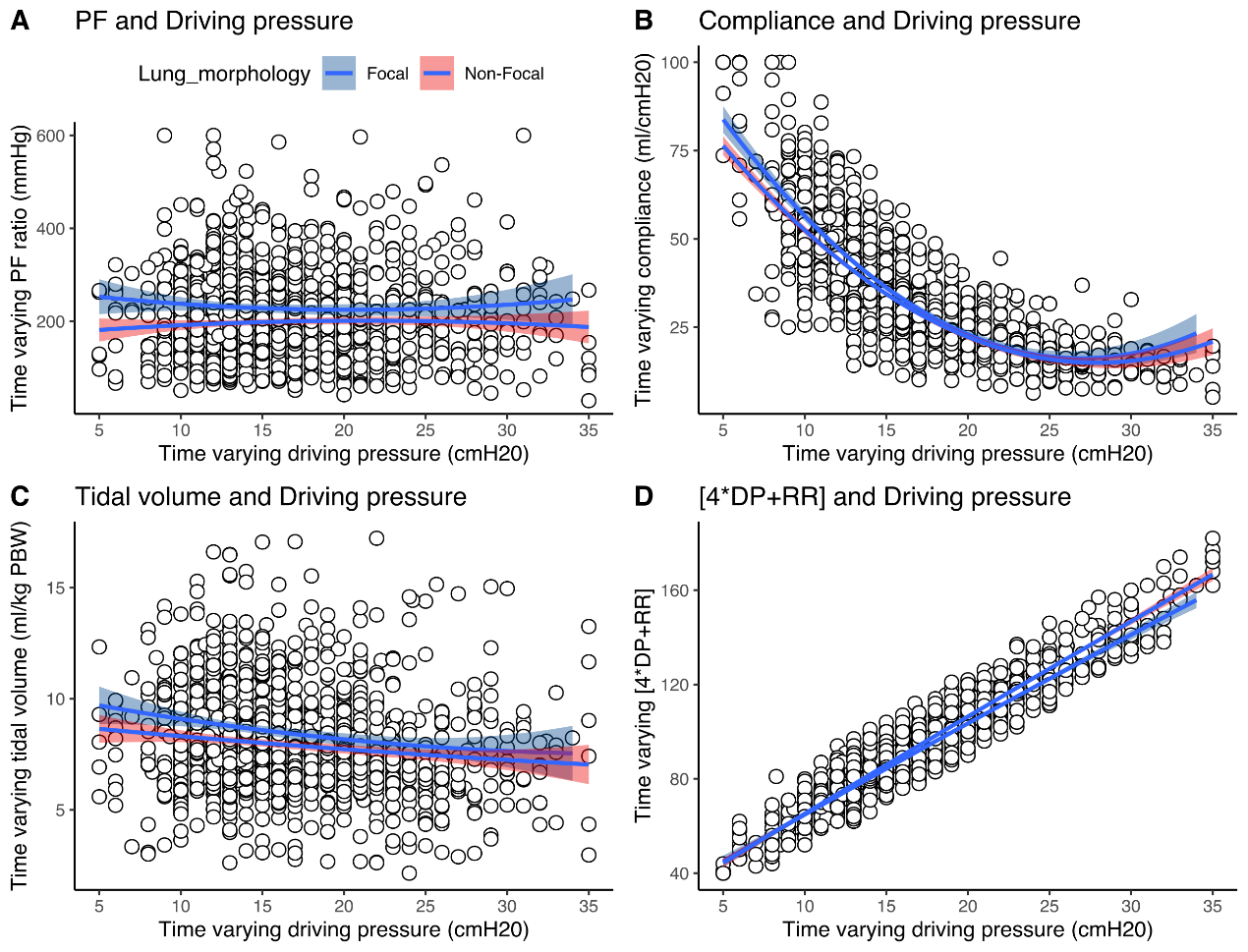


PF ratio: PaO_2_/FiO_2_ ratio; DP: Driving pressure; RR: Respiratory rate.

Figure S5: The Correlation between Mechanical power and PF ratio (A), Respiratory compliance (B), Tidal volume (C) and [(4xDP)+RR] (D) in patients with different lung morphology.


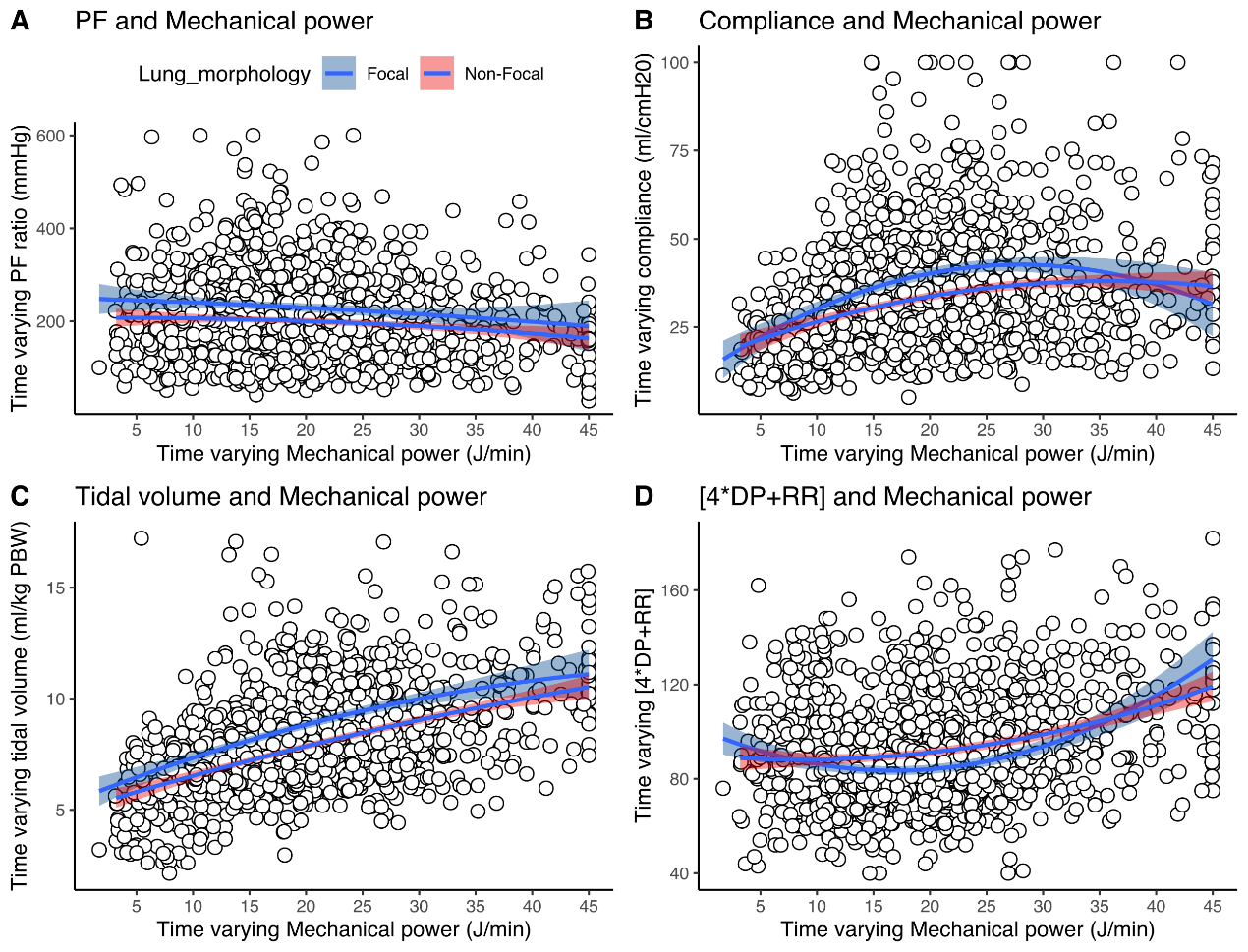


PF: PaO_2_/FiO_2_ ratio; DP: Driving pressure; RR: Respiratory rate.

Figure S6: Mortality by lung morphology in patients with ARDS.


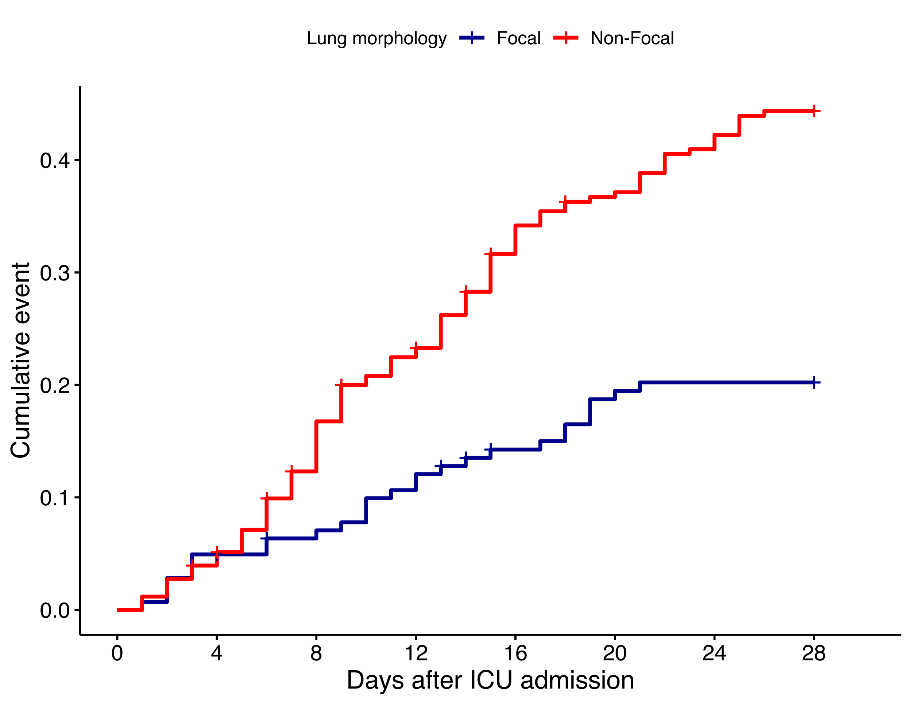


P<0.001

Figure S7: The C-index of each Cox regression Model in total patients and patients with different with lung morphology.


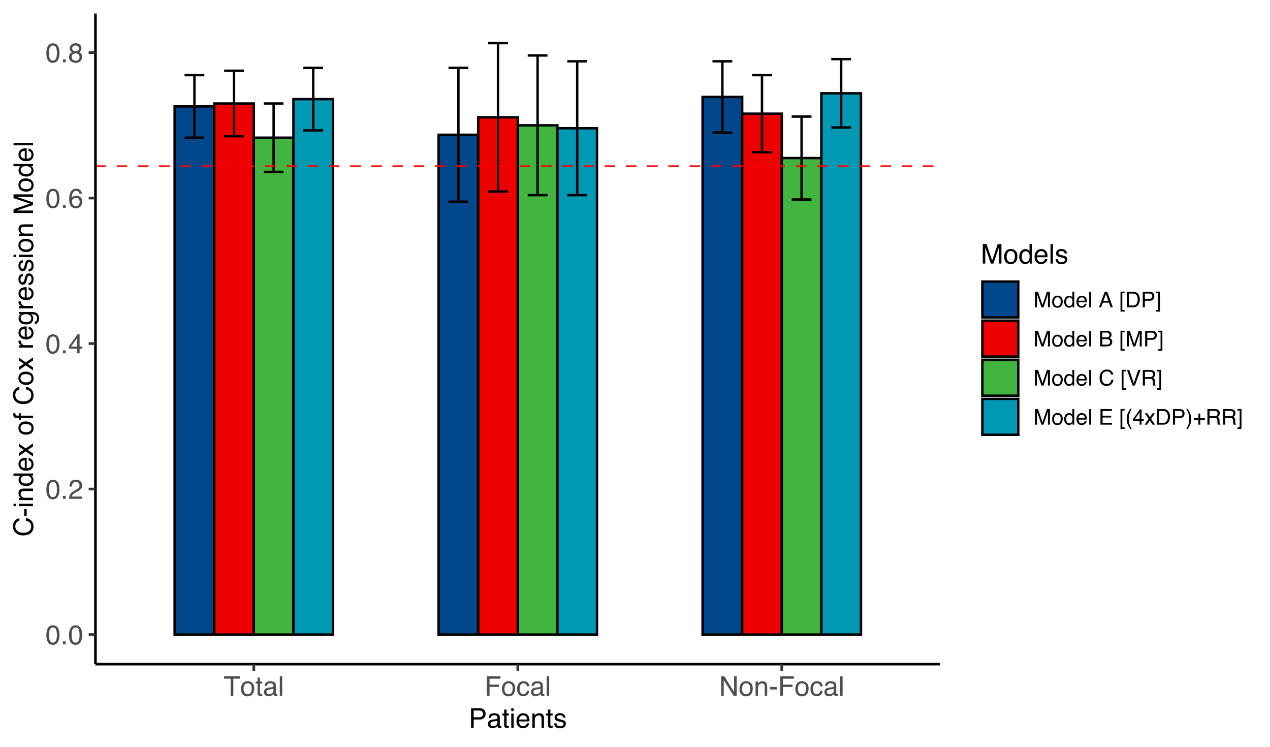


The red dashed line represents the C-index of Baseline risk model (0.644). Error bars represent 95% confidence intervals. DP: Driving pressure, MP: Mechanical power; VR: ventilatory ratio; RR: Respiratory rate.
